# Nonlinear Cellular Mechanical Behavior Adaptation to Substrate Mechanics Identified by Atomic Force Microscope

**DOI:** 10.3390/ijms19113461

**Published:** 2018-11-04

**Authors:** Keyvan Mollaeian, Yi Liu, Siyu Bi, Yifei Wang, Juan Ren, Meng Lu

**Affiliations:** 1 Department of Mechanical Engineering, Iowa State University, Ames, IA 50011, USA; keyvanm@iastate.edu (K.M.); yil1@iastate.edu (Y.L.); siyub@iastate.edu (S.B.); 2 Department of Electrical and Computer Engineering, Iowa State University, Ames, IA 50011, USA; yifeiw@iastate.edu (Y.W.); menglu@iastate.edu (M.L.)

**Keywords:** cell mechanics adaptation, substrate mechanics, Atomic Force Microscope, cytoskeleton

## Abstract

Cell–substrate interaction plays an important role in intracellular behavior and function. Adherent cell mechanics is directly regulated by the substrate mechanics. However, previous studies on the effect of substrate mechanics only focused on the stiffness relation between the substrate and the cells, and how the substrate stiffness affects the time-scale and length-scale of the cell mechanics has not yet been studied. The absence of this information directly limits the in-depth understanding of the cellular mechanotransduction process. In this study, the effect of substrate mechanics on the nonlinear biomechanical behavior of living cells was investigated using indentation-based atomic force microscopy. The mechanical properties and their nonlinearities of the cells cultured on four substrates with distinct mechanical properties were thoroughly investigated. Furthermore, the actin filament (F-actin) cytoskeleton of the cells was fluorescently stained to investigate the adaptation of F-actin cytoskeleton structure to the substrate mechanics. It was found that living cells sense and adapt to substrate mechanics: the cellular Young’s modulus, shear modulus, apparent viscosity, and their nonlinearities (mechanical property vs. measurement depth relation) were adapted to the substrates’ nonlinear mechanics. Moreover, the positive correlation between the cellular poroelasticity and the indentation remained the same regardless of the substrate stiffness nonlinearity, but was indeed more pronounced for the cells seeded on the softer substrates. Comparison of the F-actin cytoskeleton morphology confirmed that the substrate affects the cell mechanics by regulating the intracellular structure.

## 1. Introduction

Living cells are exquisitely sensitive to mechanical stimuli in their extracellular environment. Among many kinds of extracellular force stimuli, the stiffness of the underlying substrate where a cell attaches to is one of the most accessible (and widely studied) biomechanical factors in affecting cellular behavior [1,2,3]. For instance, studies have shown that mesenchymal stem cells that attach to stiffer substrates commit to an osteogenic fate [4], whereas the cells express a neurogenic phenotype when are seeded on softer substrates [5]. In addition, the stiffness of the extracellular matrix (ECM) regulates the structure, motility, and proliferation of the cells [6]. Although extensive efforts have identified multiple signaling pathways, such as downstream signaling of $\alpha_{v}\beta_{3}$ and ${\mathrm{RPTP}}_{\alpha }$ [7] and tyrosine phosphatase and kinase [8], in the cellular rigidity sensing process, how the substrate mechanics affects the cellular mechanical properties at different depths remains poorly understood. Questions such as which micro-/nano-scale cellular properties are more sensitive to the substrate mechanics and how the substrate stiffness affects the time-scale and length-scale of cellular mechanical responses have not yet been investigated. The absence of these studies directly limits in-depth understandings of cellular mechanotransduction process.

Previously, the effect of substrate mechanics on cellular mechanics has been mostly studied by quantifying the dependence of cellular stiffness (i.e., Young’s modulus) on substrate rigidity at a certain indentation depth using atomic force microscope (AFM) owing to its ultra-high spatial and force resolutions and real-time data capturing capability [9,10]. Studies have shown that cells are highly adaptive to the substrate stiffness: cell stiffness has a monotonically increasing relation with the substrate rigidity [11,12,13]. Wang et al. (2000) reported that normal NIH/3T3 cells reacted to the rigidity of the substrate with a decrease in the rate of DNA synthesis and an increase in the rate of apoptosis on flexible substrates [14]. Takai et al. (2005) found that the apparent elastic modulus of MC3T3-E1 cells were substrate dependent [15]. However, due to the biphasic nature and self-organization of living cells, stiffness alone is not adequate enough to represent the cellular mechanical and rheological behavior under various force measurement conditions [16,17]. Since cell rheology has been shown time/frequency dependent [16,17,18], cellular viscosity should also be considered when studying the effect of substrate mechanics. Moreover, as the largest portion of the cell—cytoplasm—essentially consists of both the intracellular fluid (e.g., the cytosol) and the viscoelastic network (e.g., the cytoskeleton), the above two aspects cannot account for the ubiquitous biphasic nature of the cytoplasm [16,17]. Therefore, poroelasticity which links the biomechanical behavior of the cells to structural hierarchy, intracellular fluid flow (cytosol), related volume change, and biological parameters, must be quantitatively investigated as well [19,20,21]. Poroelasticity describes the cell’s ability to equilibrate the intracellular pressure under external loading force (i.e., localized deformation) through active intracellular fluid redistribution (efflux) [16,17], and can be represented by the poroelastic diffusion coefficient, *D*, which depends on elastic modulus *E*, the pore size of the cytoskeleton meshwork ξ, and the viscosity of the cytosol μ [16,17,20,22]. Therefore, to investigate the time-scale and length-scale dependence of cell response to substrate mechanics, elasticity, viscoelasticity, and poroelasticity of the cells must be quantified simultaneously. Moreover, studies have reported that cellular mechanical behavior is nonlinear [16,17]—ascribed to the multi-layered heterogeneity of living cells. Thus, the measured mechanical behavior entirely depends on the deformation (e.g., the indentation in AFM measurements) scale of the cells, which determines the specific cell layers that are disturbed by the measurement. Therefore, the effect of substrate mechanics on the nonlinearity of cellular biomechanical behavior needs to be studied as well. However, such an important aspect has not been reported yet in previous studies.

In this study, we investigated the effects of substrate’s mechanics on the nonlinear mechanical behavior of living cells using AFM force indentation measurements. As studies have shown that the cell–substrate relation in terms of mechanical properties may change significantly based on the cell type, two different cell lines were studied: an epithelial cell line (Madin–Darby canine kidney (MDCK) and a fibroblast cell line (NIH/3T3)). Specifically, for each cell type, the cells were cultured on substrates with different stiffness (Polydimethylsiloxane (PDMS) with the base-to-curing agent ratios of 10:0.5, 10:1, and 10:3, and the polystyrene cell culture dish), and the relation between the substrate mechanics and cell nonlinear mechanical behavior (stiffness, viscosity, and poroelasticity) was investigated by indenting the cells at different depths. Moreover, to understand how the substrate affects the cellular mechanics, the cells were fluorescently stained to study the actin filament (F-actin) morphology change caused by the four substrates.

## 2. Results and Discussion

Although previous studies have shown the effect of the substrate stiffness on the stiffness of living cells [15,23], more detailed results on how the substrate mechanics affects the cell rheology and its nonlinearity has not been reported. Thus, we investigated the effect of the four substrates with different stiffness (three PDMS substrates and polystyrene cell culture dish) on the elasticity, viscoelasticity, and poroelasticity of NIH/3T3 and MDCK cells. The cell Young’s modulus, *E*, was quantified according to the Hertz contact model [24,25] (Equation (2)) using the data obtained at the end of the indenting process. Then, cell viscoelastic and poroelastic behavior were quantified using the force and indentation data of the force–relaxation process (see Equations (7) and (9)).

### 2.1. Effect of Substrate Mechanics on the Nonlinear Elastic and Viscoelastic Behavior of the Cells

**Figure 1:**
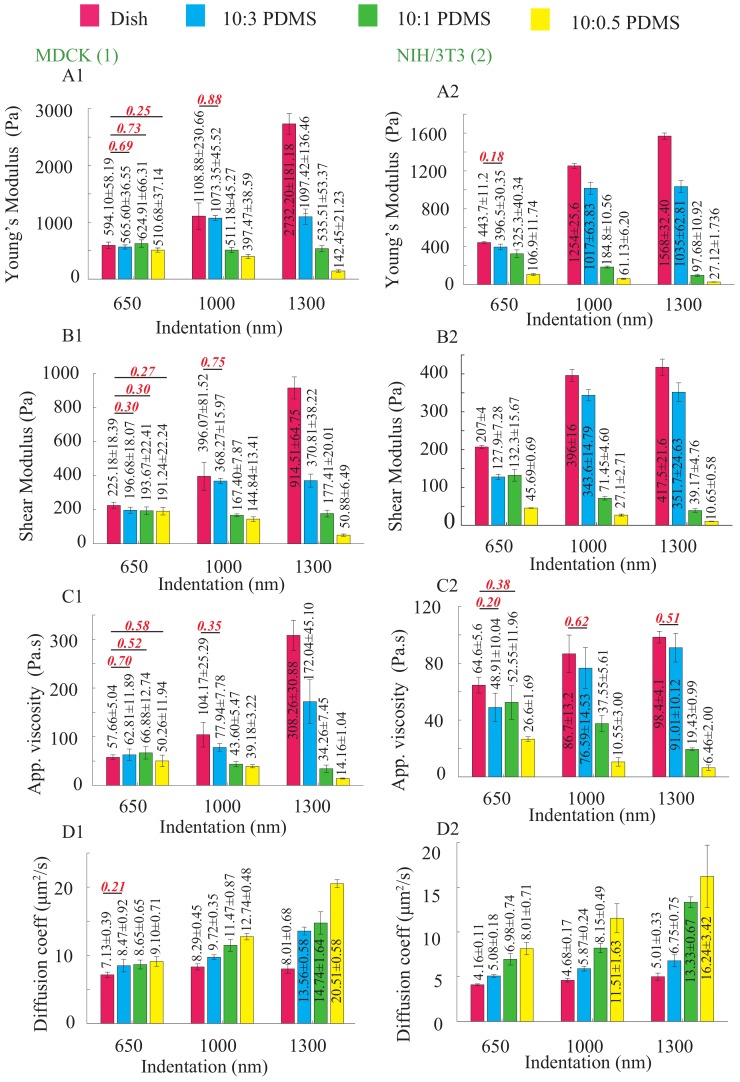
The nonlinear cellular: (**A1**,**A2**) Young’s modulus; (**B1**,**B2**) shear modulus; (**C1**,**C2**) apparent viscosity; and (**D1**,**D2**) diffusion coefficient of MDCK and NIH/3T3 cells seeded on different substrate, respectively, quantified at different indentation depths at the indenting velocity of 20 μm/s. The AFM measurements were performed on six different cells at each indentation depth and the error bars represent the standard errors. *n* = 6. Student’s *t*-test was performed to analyze the statistical difference: for each indentation, data were compared with respect to the ones measured on the dish (control) at the same indentation; and for each substrate, the data measured at the minimum indentation (650 nm) for that substrate were chosen as control. A *p* < 0.05 was yielded for each comparison, unless otherwise denoted in the figure (with *p* values in red bold italic font).

**Figure 2:**
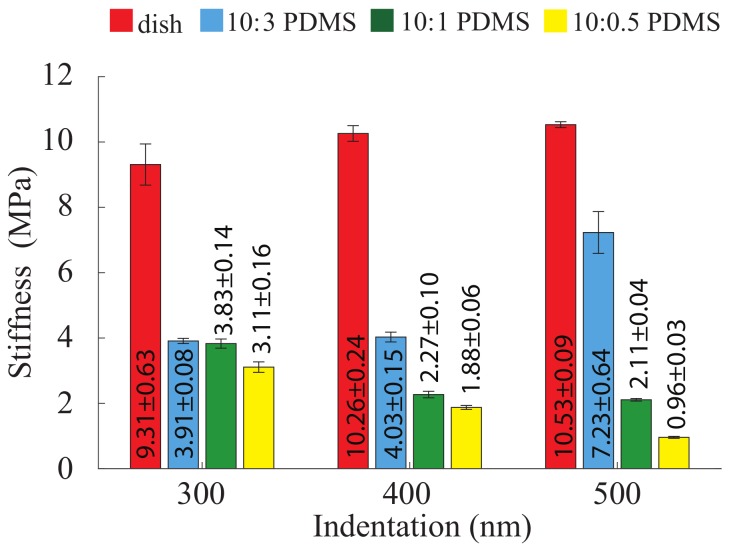
Stiffness nonlinearity of the four different substrates measured at the indenting velocity of 20 μm/s. The error bars represent the standard errors. *n* = 6. Student’s t-test was performed to analyze the statistical difference: for each indentation, data were compared with respect to the ones measured on the dish (control) at the same indentation; and for each substrate, the data measured at the minimum indentation (650 nm) for that substrate were chosen as control. A *p* < 0.05 was yielded for each comparison unless otherwise denoted in the figure (with *p* values in red bold italic font).

The results clearly show that the cellular mechanical behavior (in terms of elasticity) is significantly different for each substrate, as shown in Figure 1. To demonstrate the substrate mechanics effect, the nonlinear stiffness (i.e., Young’s modulus) of the four substrates was also measured (20 μm/s, see Figure 2). Comparing Figure 1 and Figure 2, it is clear that the mechanical behaviors of both NIH/3T3 and MDCK cells adapt to the substrates mechanics closely, including both the stiffness and its nonlinearity. Note that, since the substrate stiffness is at least three orders higher than the cells’, and the indentation used was less than one quarter of the cell height, substrate effect could be ignored during the cell mechanics quantification. Thus, the quantified results in Figure 1 indeed represent the biomechanical behavior of the measured cells.

Significant changes are shown for the elasticity (Young’s modulus *E* and shear modulus *G*) and viscoelasticity (apparent viscosity η) of both types of cells seeded on the four substrates. In general, the cell elasticity and viscoelasticity are synchronized with the substrate stiffness closely, as shown in Figure 1. At each indentation depth, *E*, *G*, and η are positively correlated with the substrate stiffness, except no clear trend is shown for MDCK cells at the lowest indentation depth. For the cells seeded on each of the four substrates, the nonlinearity of these three cellular mechanical parameters (*E*, *G*, and η) are consistent with the substrate stiffness nonlinearity as well. Specifically, as the stiffness of the 10:3 PDMS and the cell culture dish is monotonic with the indentation depth (see Figure 2), *E* of NIH/3T3 and MDCK cells on 10:3 PDMS increased by 161% and 94%, respectively, when the indentation was increased from 650 to 1300 nm, and the increase was 253% and 360%, respectively, for the cells seeded on the culture dish. However, on the two softer substrates, which become softer as the indentation depth increases, the Young’s modulus of NIH/3T3 and MDCK cells on 10:0.5 PDMS at the indentation depth of 1300 nm was at most 27 and 142 Pa, respectively—more than 70% reduction compared to the values at the 650 nm indentation. In addition, *E* reduced at least 14% for both cell types on 10:1 PDMS when the indentation depth was doubled from 650 nm. Similar changes of nonlinearity of the shear modulus and apparent viscosity were also observed for both cell types. As shown in Figure 1, the shear modulus and the apparent viscosity of NIH/3T3 and MDCK cells are synchronized with the substrate stiffness at each indentation depth, respectively. Specifically, *G* and η for these two types of cells on the cell culture dish and 10:3 PDMS increased by at least 89% and 52%, respectively, when the indentation depth increased from 650 to 1300 nm. However, both decreased for both cell types on the softer substrates (i.e., 10:1 and 10:0.5 PDMSs).

**Figure 3:**
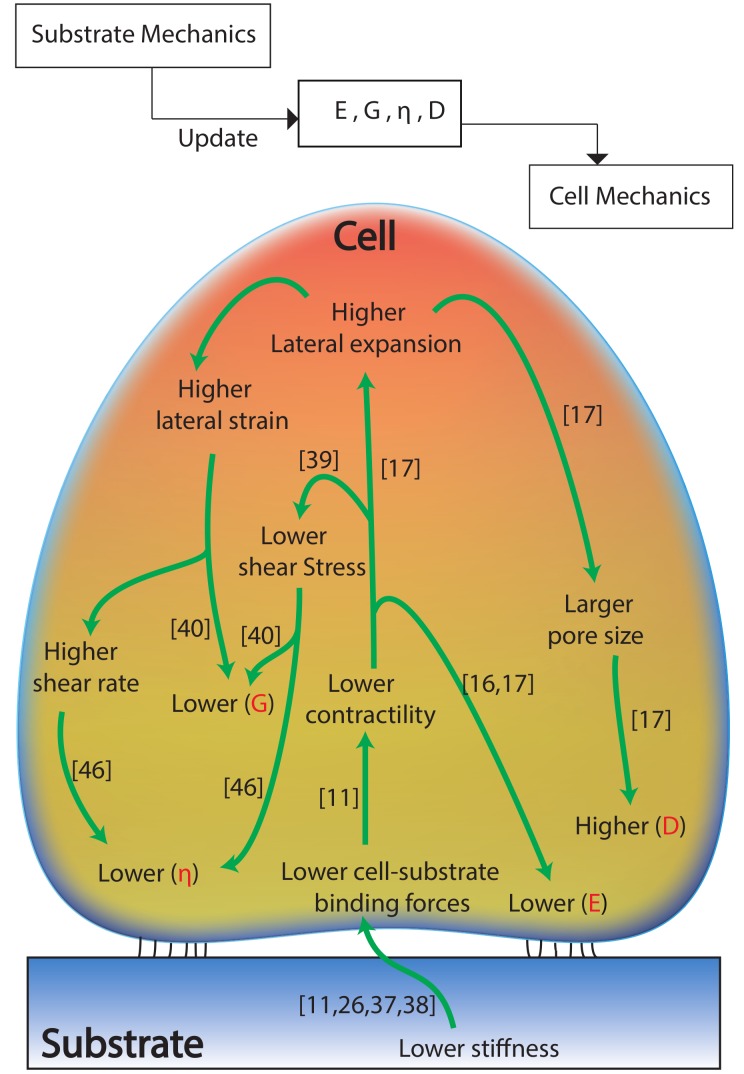
Schematic sketch of the cell biomechanical behavior change in response to substrate mechanics.

The experiment results demonstrated that the adherent cells sense and adapt to substrate mechanics: the nonlinear cellular elasticity and viscosity are regulated by the substrate stiffness nonlinearity. However, previous studies only showed the dependence of the cellular elasticity to the substrate stiffness at a single measurement depth [23]. To explain the presented results on the adaptation of the cellular biomechanical behavior to the substrate’s nonlinear mechanics, a systematic sketch to illustrate the cell–substrate contact mechanism was generated based on the previous studies on cell–substrate interaction and is presented in Figure 3. Specifically, as reported previously, in response to a stiffer substrate, stronger cell–substrate bonding is established (i.e., larger cell–substrate adhesion force) [26,27,28], which further leads to the stiffening of the cells—higher *E* [23]. Thus, at each measured indentation depth, the cell Young’s modulus is positively correlated with the substrate stiffness. By changing the elastomer base-to-curing agent ratio from 10:0.5 to 10:3, the cross-linking density of the PDMS substrates increased significantly [29], which further stiffens the polymer network (see Figure 2). For the harder substrates (10:3 PDMS and polystyrene cell culture dish), the highly cross-linked polymer network generates stronger resistance at deeper layers from the surface, thus a higher stiffness is yielded as the indentation depth increases [29,30,31]. However, for the softer PDMSs (10:1 and 10:0.5), not only the stiffness at a certain indentation depth is lower, but also the polymer behaves softer at the deeper indentation [32,33]. This is because low cross-linking degree makes the effect of higher order displacement gradients more pronounced due to higher molecular motion freedom at deeper indentations [29,31]. As the indentation depth increases, the subcellular cell–substrate interface (i.e., focal adhesion) is compressed further, thus the stiffness of the substrate at deeper layer is sensed and adapted by the cells (see Figure 3). Therefore, similar Young’s modulus nonlinearity was observed for the cells. Note that the results presented are not contradictory to the previous finding that the cell stiffness measured on glass coverslips at nanometer scale decreases with the indentation increase [34]. Indeed, the cell mechanical behavior quantified at micrometer scale is quite different from that measured at nanometer scale, as the former leads to a “bulk” scale characterization and the latter is localized quantification. This difference can be directly seen from the quantified cell Young’s modulus values: *E* is at the order of 10^2^ Pa in this study and previous work where μm sized probes were used [17,35]; however, *E* is at the order of kPa when nm sized probes were used [16,36]. In fact, our results agree with the previous studies on cell elasticity nonlinearity well: it has been reported that the Young’a modulus of cells seeded on glass coverslips increased as the indentation depth increased from 300 to 1000 nm [35].

At the same time, as the cell–substrate bonding strength is monotonically increasing with the substrate stiffness [11], the softer the substrate is, the weaker the cell–substrate adhesion force is [26,37,38]. This directly results in lower shear stress of the cytoskeleton and lower cell contractility [11,39]. Thus, the cells are prone to the higher degree of lateral expansion once they are indented at a certain depth [11,17], which directly leads to the larger shear strain. Thus, it appears that the cells possess lower shear modulus, (*G* = shear stress/shear strain [40]) when their substrate is softer. In addition, the higher degree of lateral expansion during indentation can cause significant expanded cytoskeleton network [17,41,42,43,44], thus the intracellular fluid flow rate (i.e., shear rate [45]) is increased. Together with the decreased cytoskeleton shear stress, the cell apparent viscosity, η = shear stress/shear rate [46], is decreased. Therefore, the cell shear modulus and apparent viscosity are also positively correlated with the substrate stiffness and its nonlinearity.

### 2.2. Effect of Substrate Mechanics on the Poroelastic Behavior of the Cells

As can be seen in Figure 1, at each measured indentation, the diffusion coefficient, *D*, is negatively correlated with the substrate stiffness for both NIH/3T3 and MDCK cells. Specifically, for all of the three measured indentation depths, the stiffer the substrate is, the lower the poroelastic diffusion coefficient is. As aforementioned, the cells are subject to larger shear strain on softer substrates due to weakened cell–substrate bonding. This indicates that the cell structure (e.g., the cytoskeleton) is more expanded on softer substrates, which directly results in larger pore radius, ξ, of the cytoskeleton network (Figure 3). Thus, larger *D* is quantified for the cells seeded on the softer substrates at each indentation, although the Young’s modulus of these cells are lower than those seeded on the harder substrates. Note that this observation is not contradictory to the poroelasticity scale law, $D∼E\xi^{2}/\mu$ (where μ is the viscosity of the cytosol), instead it concurs with the previous findings that the pore radius is more dominant than *E* in affecting the cell poroelasticity [16,17]. However, the nonlinearity of cell poroelasticity did not show a unanimous relation with the substrate stiffness nonlinearity. Specifically, when the indentation depth was doubled from 650 nm (see Figure 1), *D* increased by 20%, 33%, 91%, and 103% for NIH/3T3 cells seeded on the cell culture dish, 10:3, 10:1, and 10:0.5 PDMSs, respectively, and 12%, 60%, 70%, and 125% for MDCK cells, respectively. However, the stiffness vs. indentation relation of the substrates were divided: monotonic for dish and 10:3 PDMS and opposite for 10:1 and 10:0.5 PDMSs, as shown in Figure 2. Note that the stiffness of 10:0.5 PDMS decreased the most (69%) compare to the other three as the indentation depth increased (see Figure 2). Therefore, the nonlinearity of the cell poroelasticity is more significant on the softer substrates, especially the one whose stiffness is the most negatively correlated with the indentation depth. When the indentation increases, the increase of ξ is more significant on the softer substrate because the weaker cell–substrate bonding can cause further cytoskeleton expansion [41,42,43,44]. In this case, even if the cells are softer (i.e., with lower *E*), *D* still increases—ξ is more dominant than *E* in affecting *D* [16,17]. As a result, the monotonic *D* vs. indentation relation for cells is more pronounced on the substrates with inversely correlated stiffness vs. indentation, and the more dramatic this inverse correlation is, the more significant cell poroelasticity nonlinearity is. Therefore, the cellular poroelasticity and its nonlinearity are also directly affected by the substrate stiffness and its nonlinearity. Note that the differences of *E*, *G*, η, and *D*, respectively, of the MDCK cells on the four substrates are less significant at the indentation depth of 650 nm compare to the other two depths and the results for NIH/3T3 cells. One possible explanation is the cell morphology difference: MDCK cells are in general much taller than NIH/3T3 cells (8 μm vs. 6 μm) and have thicker plasma membrane as epithelial cells [47], thus the substrate effect on the MDCK cell behavior at the low indentation (e.g., 650 nm) was not as significant as that on the NIH/3T3 cell. Once the MDCK cells are indented deep enough (i.e., deeper layers of cells are probed), the effect of substrate mechanics becomes more pronounced and both the Young’s modulus and the shear modulus follow the same trend compared to the substrate stiffness. To further understand how the cells sense the substrate mechanics, we also investigated the F-actin cytoskeleton for the MDCK and NIH/3T3 cells seeded on the four different substrates.

### 2.3. Substrate Mechanics Affects Cell Biomechanical Behavior by Regulating the Cell Morphology

**Figure 4:**
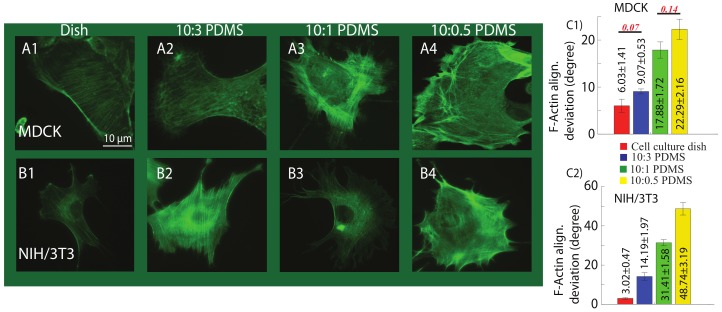
Examples of F-actin cytoskeleton images of (**A1**–**A4**) MDCK and (**B1**–**B4**) NIH/3T3 cells seeded on the four substrates, respectively. The cells were fixed and stained 24 hours after being seeded. Scale bar: 10 μm. The F-actin alignment deviations for (**C1**) MDCK and (**C2**) NIH/3T3 cells seeded on each substrate. *n* = 6. Student’s t-test was performed to analyze the statistical difference: data for all substrates were compared to each other for each cell type. A *p* < 0.005 was yielded for each comparison unless otherwise denoted in the figure (with *p* values in red bold italic font).

In this study, the effect of the substrate mechanics on F-actin distribution as a sensory mechanism of the cell was investigated [48]. The differences of F-actin structure were clearly observed on the four substrates, as shown in Figure 4. Comparing the F-actin alignment deviation for cells on each substrate, it is clear that stiffer substrate led to more uniform F-actin organization (i.e., more uniformly distributed F-actin alignment angles) of both MDCK and NIH/3T3 cells; however, F-actin was disoriented on soft PDMSs, and the softer the substrate was, the less uniform the F-actin alignment was. This observation is consistent with previous findings that stronger actin-myosin cross bridging on harder substrates can lead to more stabilized and enhanced F-actin cytoskeleton alignment [11].

Combine with the significant biomechanical behavior differences of the cells on the four substrates, it is clear that the substrate mechanics affects the cellular biomechanical behavior through regulating the inner structure, such as F-actin cytoskeleton. Specifically, our previous work [16] has shown that depolymerization of F-actin would cause significant reduction of the cell stiffness and increase of the cell diffusion coefficient, and the nonlinearities of cell stiffness and poroelasticity became more significant as well. In addition, it has been reported that depolymerization of F-actin contributes to the reduction of cytoskeleton stiffness and the increase of the cytoplasmic pore size [16,17,35]. Thus, the measured cell mechanical behavior on different substrates, which caused F-actin structure change, associated to depolymerized F-actin. Moreover, as previously reported, living cells respond to the substrate stiffness by reformation of the cytoskeleton components and adhesion molecules activities [49,50]. Particularly, the stimulation of cytoskeleton effectors including Ras superfamily proteins leads to enhanced stress fibers and increases cell growth on stiffer substrates [51,52]. Tyrosine phosphorylation, calmodulin and vinculin activated myosin, and enhanced Rho and Rac proteins activity along with stronger actin-myosin cross-bridging on the harder substrates lead to more stabilized local adhesion and enhanced F-actin cytoskeleton alignment [51,52,53,54,55]. Therefore, the cell–substrate bonding strength—a direct result of substrate stiffness—modifies the cytoskeleton integrity and thus regulates the cell mechanical behavior. Specifically, weaker cell-exerted forces to the softer substrate in response to mechanics of the substrate leads to cytoskeleton and stress fibers deformation, lower tyrosine phosphorylation, calmodulin and vinculin activities, which further causes lower contractility of the cell and weaker cross-bridging of the actin-myosin [51,52,53,54,55]. Thus, lower Young’s modulus was quantified. In addition, the lower cell–substrate interaction and instability of the focal adhesion on the soft polymers causes weakened (less uniformly aligned) cell cytoskeleton, resulting in increased intracellular fluid flow and thus higher diffusion coefficient. This enhanced intracellular fluid flow also contributes to the reduction of apparent viscosity of the cells on the softer substrates.

Therefore, the results confirmed that substrate mechanics regulates cellular biomechanical behavior by modifying the cytoskeleton structure. These findings on the adaptation of the biomechanical behavior of the adherent cells to the substrate mechanics may be further used to control and regulate the cellular mechanical behavior to manipulate the mechanotransduction process. To fully understand and model the cell–substrate mechanical sensing, more in-depth investigations are needed to explain the physiological and biomechanical behavior of the cells caused by different extracellular environment. As for the future work, it is of importance to study the cell morphology variation (e.g., cell shape) caused by substrate mechanics. As the lower stiffness regime (i.e., <100 kPa) of substrate stiffness is more relevant to cell differentiation and organization of the cytoskeleton, the proposed work will be extended to this regime as well. Furthermore, as 3D cell culture environment is more similar to the actual cell existing condition in living bodies and the cell mechanical behavior is quite different from 2D culture cases [56,57,58], the study of cell mechanical behavior change due to culture environment change will be further extended to 3D cases in the future.

## 3. Conclusions

In this study, the effect of substrate mechanics on biomechanical behavior of the cells was investigated using AFM indentation approach. The elastic, viscoelastic, and poroelastic nonlinearity of MDCK and NIH/3T3 cells on substrates with different mechanics (i.e., 10:0.5, 10:1, 10:3 PDMSs, and polystyrene cell culture dish) were quantified at different indentation depths. It was found that the cell elasticity, viscoelasticity, and their nonlinearities were synchronized with the substrate stiffness and its nonlinearity, respectively. The diffusion coefficient of the cells increased, monotonically, with the increase of the indentation depth on all substrates. Particularly, this poroelasticity nonlinearity was more pronounced for the cells cultured on the softer substrates due to larger lateral expansion of the cell and larger cytoskeletal pore size. Moreover, the cell F-actin cytoskeleton images suggested that the stiffer the substrate was, the more uniform the F-actin alignment was. Thus, combining the results together, it is clear that the substrate mechanics affects the cellular mechanics by regulating the inner structure of the cells.

## 4. Materials and Methods

### 4.1. Chemicals

Sylgard 184 silicone elastomer and elastomer base were purchased from Ellsworth (Germantown, WI, USA). Dulbecco’s Modified Eagles Medium (DMEM) was purchased from Sigma Aldrich (St. Louis, MO, USA). Minimum Essential Medium Eagle (MEM) and Phosphate-Buffered Saline (PBS) were purchased from Corning cellgro (Manassas, VA, USA). Fetal bovine Serum (FBS) and penicillin–streptomycin (pen-strep) were obtained from Gibco (Grand Island, NY, USA). Paraformaldehyde (PFA, 4% in PBS) was purchased from Alfa Aesar (Ward Hill, MA, USA). Bovine Calf Serum (BCS) was purchased from VWR (Radnor, PA, USA). Triton X-100 was purchased from Fisher Scientific (Fair Lawn, NJ, USA). Acti-stain^™^ 488 Phalloidin was purchased from Cytoskeleton, Inc. (Denver, CO, USA).

### 4.2. Polymer Substrate Preparation

To prepare PDMS substrates with different stiffness, Sylgard 184 silicone elastomer base and the curing agent with the base-to-curing agent ratios of 10:0.5, 10:1, and 10:3 were mixed for around 10 min. The mixtures were degassed under vacuum until air bubbles disappeared (around 30 min) and poured onto flat polystyrene Petri dishes. The thickness of the prepolymers was kept constant as 2 mm. Then, the prepolymers were cured at 70 °C for 10 h following cooling to room temperature (25 °C).

### 4.3. Cell Culture and Treatment

NIH/3T3 cells were cultured in DMEM containing 10% BCS and 1% pen-strep. MDCK cells were cultured in MEM containing 10% FBS and 1% pen-strep. The cells were subcultured at a density of $1.0\times 10^{4}$ cells/mL on the three PDMS substrates and polystyrene cell culture dishes (35 mm Falcon, Durham, NC, USA) and maintained at 37 °C in 5% CO_2_ incubator for 24 h prior to the AFM measurement. For the AFM nanomechanical measurements, the existing medium in the dishes was replaced by fresh growth medium to remove dead and loosely attached cells.

### 4.4. Immunofluorescence and F-Actin Quantification

To capture the F-actin cytoskeleton images, cell growth medium was removed from the dish following washing the cells with PBS at 37 °C to remove the dead and loosely attached cells. Then, the cells were fixed using 4% PFA/PBS and kept at room temperature for 10 min. The cells were then permeabilized for 5 min at room temperature using 0.5% Triton X-100 in PBS. Finally, after rinsing the cells with PBS three times, the actin cytoskeleton was stained using Actin-stain^™^ 488 Phalloidin at a final concentration of 100 nM in PBS, and the cells were kept in dark for 30 min at room temperature. Then, the fluorescent F-actin cytoskeleton images were obtained using an inverted optical microscope (Olympus, IX73, Japan) and equipped with a sola light engine (Lumencor, Beaverton, OR, USA) offering access to solid state illumination. At least eight images were taken per substrate for each cell type.

The F-actin alignment deviation was quantified using MATLAB image processing tool. F-actin fibers were detected using Canny edge detection in this program. Then, the F-actin alignment deviation was then quantified by calculating the variance in the fiber orientation angles, as determined using Hough transform.

### 4.5. Atomic Force Microscopy (AFM) Measurement

**Figure 5:**
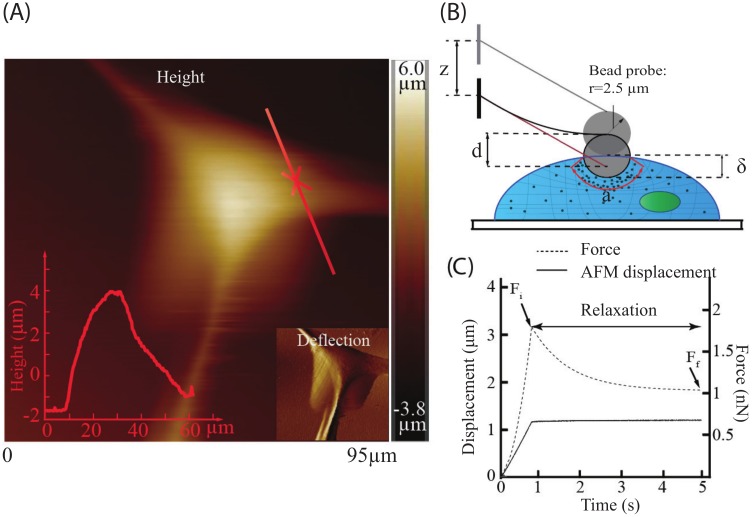
(**A**) AFM topography image of an MDCK cell, where the red cross represents the measurement site. (**B**) AFM measurement of the cells with a sphere probe (radius 2.5 μm), where the area in red represent the probe–cell contact size. (**C**) The probe–cell interaction force and AFM displacement (*z*) profile during the force–relaxation process, where $F_{i}$ is the probe–cell interaction force at the beginning of the relaxation process (i.e., the end of the indenting process), and $F_{f}$ is the force at the end of the relaxation process.

All AFM measurements were performed at room temperature in cell growth medium with a Bruker BioScope Resolve AFM system (Santa Barbara, CA, USA), which is integrated with an inverted optical microscope (Olympus, IX73, Japan). Colloidal AFM probe (Novascan, IA, USA) with sphere radius of 2.5 μm was used. The cantilever spring constant of 0.02 N/m was acquired using thermal tune approach [59]. Drive voltage and sensor data of the AFM system were acquired using an NI PCIe-6353 DAQ board (National instrument, Austin, TX, USA) with Matlab Simulink Desktop Real-time platform (Mathworks, MA, USA). Cells were measured at a location away from the top to minimize the nucleus effect (see Figure 5A). The height of the measured NIH/3T3 and MDCK cells are 7 ± 1 μm and 8.5 ± 1.5 μm, respectively (mean ± standard deviation). To minimize the effect of the finite cell thickness and substrate effect [60,61,62], the target indentation depths were chosen as 650, 1000, and 1300 nm which were less than a quarter of the minimum cell height. To investigate the effect of substrate mechanics on the biomechanical behavior of the MDCK and NIH/3T3 cells, the AFM indenting speed was kept at 20 μm/s until desired indentations were reached (i.e., the indenting process), and then the probe was kept resting on the cell at that position for five seconds (i.e., force–relaxation process) (see Figure 5B,C). The indenting velocity and indentation depths were chosen based on previous studies [16,35,63] to observe the cellular poroelastic force relaxation by triggering different layers of the cells. For the two types of cells seeded on each substrate (three PDMS ones and polystyrene cell culture dish), the AFM measurement was performed on at least six different cells at each indentation depth.

### 4.6. Biomechanical Quantification of the Cells

#### 4.6.1. Cell Elasticity

Indentation depth, δ(t), was obtained using the cantilever deflection, d(t), and the AFM displacement, z(t), as [64]

(1) δ(t)=z(t)−d(t).

Since the AFM probe used was spherical and the cells were indented at a speed (20 μm/s) that was faster than the intracellular fluid efflux rate (1.5–8.5 μm/s) [16,17], the cells could be treated as an incompressible material during the indenting process [17,65] and the cell Young’s Modulus was then quantified using the Hertzian contact model [24,66], i.e.,

(2) $$F\left(t\right)=\frac{4}{3}\frac{E}{1-{\bar{\nu }}^{2}}r^{\frac{1}{2}}\delta^{\frac{3}{2}}\left(t\right),$$

where F(t)=k×d(t) is the probe–cell interaction force (*k* is the spring constant of the cantilever), *r* = 2.5 μm is the sphere probe radius, and $\bar{\nu }$ = 0.5 is the incompressible cell Poisson’s ratio. The shear modulus of the undrained (i.e., incompressible during the fast indenting process) cell network, *G*, was then calculated through [24]:

(3) $$F_{i}=\frac{16}{3}Ga\bar{\delta },$$

where $\bar{\delta }$ and $F_{i}$ are the indentation depth and probe–cell interaction force at the beginning of the force–relaxation process (i.e., the end of the fast indenting process), respectively. The probe–cell contact size, *a*, was quantified using the probe radius and the indentation depth as

(4) $$a=\sqrt{r\bar{\delta }}.$$

During the force–relaxation process (i.e., probe resting on the cells after fast indenting once the targeted indentation depth was reached), significant intracellular efflux occurs to equilibrate the unbalanced inner pressure of the cell (caused by fast indentation)—cell poroelasticity [17,67]. As a result, the probe–cell interaction force decreases significantly even when the AFM displacement was kept unchanged. The fully relaxed force (i.e., the probe–cell interaction force at the end of the relaxation process (e.g., five seconds after fast indenting)), $F_{f}$, could be quantified as [17]:

(5) $$F_{f}=\frac{8}{3(\;1-\nu )\;}Ga\bar{\delta }$$

where ν denotes the cell Poisson’s ratio during the force–relaxation process. Note that ν is different from $\bar{\nu }$ as the cells are compressible due to the intracellular fluid efflux during the relaxation process. Therefore, the Poisson’s ratio of the solid cellular matrix during the relaxation process could then be quantified using Equations (3) and (5) as:

(6) $$\nu =1-\frac{F_{i}}{2F_{f}}$$

#### 4.6.2. Viscoelasticity

With the Poisson’s ratio quantified using Equation (6), the viscoelastic behavior of the cells was obtained following the method proposed by Darling et al. [68]:

(7) $$F\left(t\right)=\frac{4}{3}\frac{E_{r}}{1-\nu }r^{\frac{1}{2}}{\bar{\delta }}^{\frac{3}{2}}\left[\;1+\left(\;\frac{\tau_{\sigma }-\tau_{\epsilon }}{\tau_{\epsilon }}\right)\;e^{-\frac{t}{\tau_{\epsilon }}}\right].$$

where $\tau_{\sigma }$ and $\tau_{\epsilon }$ are the relaxation time constants for load and deformation, respectively. $E_{r}$ is the relaxed modulus. The values of $E_{r}$, $\tau_{\sigma }$, and $\tau_{\epsilon }$ were obtained by fitting the force–time curve during the relaxation process using Equation (7), and the apparent viscosity of the cell was then approximated as [69]

(8) $$\eta =E_{r}\left(\;\tau_{\sigma }-\tau_{\epsilon }\right)\;.$$

#### 4.6.3. Poroelasticity

Since the cell size (>30 μm) was more than ten times larger than the AFM tip radius (2.5 μm), the probe–cell interaction could be approximated as a poroelastic half-space indented by a spherical indenter, and the following empirical poroelastic model obtained by finite-element-analysis was used for analyzing the cell poroelasticity [70]:

(9) $$\frac{F\left(t\right)-F_{f}}{F_{i}-F_{f}}=0.491e^{-0.908\sqrt{\frac{Dt}{a^{2}}}}+0.509e^{-1.679\frac{Dt}{a^{2}}}.$$

where *D* is the diffusion coefficient, and was obtained by fitting the force–time curve during the relaxation process using Equation (9).

### 4.7. Stiffness Quantification of the Substrates

The Young’s moduli of the cell culture dish and the PDMS substrates with different base to agent ratio (i.e., 10:0.5, 10:1, and 10:3) were measured in air using AFM. The AFM indenting speed was kept at 20 μm/s until the desired indentation depths (300, 400, and 500 nm) were reached and then the probe was returned to its original position. Since the substrates are much harder than living cells, a stiffer AFM cantilever—TAP150A (Santa Barbara, CA, USA)—with the conical radius and the spring constant of 8 nm and 5 N/m, respectively, was used for polymer characterization. The substrate stiffness was then quantified as following [24]

(10) $$F_{t}=\frac{2}{\pi }\tan\left(\alpha \right)\frac{E_{t}}{1-\nu_{t}^{2}}\delta_{t}^{2}\left(t\right).$$

where α and $\nu_{t}$ are the tip opening angle and the Poisson ratio of the substrates, respectively. Additionally, the Poisson’s ratio $\nu_{t}=0.5$ [17] was used for elasticity measurements. $F_{t}$ is the tip–substrate interaction force, and $E_{t}$ denotes the substrate stiffness.

### 4.8. Curve Fitting and Statistical Analysis

Collected force–time relaxation curves from AFM were fitted by the poroelastic (Equation (9)) and viscoelastic model (Equation (7)) and the RMS fitting error was calculated to ensure the measurement consistency. Data in figures are presented as mean ± standard error. Student’s t-test was performed to evaluate statistical significance, and the returned *p* values were reported in the figures.
